# Establishment of Prognostic Signatures of N6-Methyladenosine-Related lncRNAs and Their Potential Functions in Hepatocellular Carcinoma Patients

**DOI:** 10.3389/fonc.2022.865917

**Published:** 2022-06-06

**Authors:** Xianbin Wu, Zhejun Deng, Xiaomin Liao, Xianxian Ruan, Nanfang Qu, Lixing Pang, Xiaoyan Shi, Shanyu Qin, Haixing Jiang

**Affiliations:** ^1^ Department of Gastroenterology, The First Afliated Hospital of Guangxi Medical University, Nanning, China; ^2^ Department of Gastroenterology, The Third Affifiliated Hospital of Guangxi Medical University, Nanning, China; ^3^ Department of Gastroenterology, Affiliated Hospital of Guilin Medical University, Guilin, China

**Keywords:** hepatocellular carcinoma, M6A, lncRNA, prognostic signature, AC012073.1

## Abstract

N6-methyladenosine (m6a)-related mRNAs and lncRNAs have been explored for their functions in several cancers. The present study aimed to identify potential signatures of m6a-related lncRNAs in hepatocellular carcinoma (HCC). We downloaded the expression and clinical data from The Cancer Genome Atlas (TCGA) database. The interacted mRNAs and lncRNAs, prognosis-related lncRNAs, potential metabolic pathways of lncRNAs, immune infiltration of various cells, and CD274 (PD-L1) -related lncRNAs were analyzed. Then, *in vitro* experiments explored the role of AC012073.1 (LOC105377626) in HCC cell lines. We found that candidate 14 lncRNA signatures play functions in HCC maybe by affecting immune infiltration, cell cycle, Notch signaling pathway, etc. LncRNA AC012073.1 (LOC105377626) functions as oncogenic roles in affecting HCC prognosis.

## Introduction

Ranking as the second malignant tumors, hepatocellular carcinoma (HCC) is the most common type of primary liver cancer, with over 700,000 patient deaths each year (1). It is estimated that primary liver cancer caused roughly 906,000 new cases and 830,000 deaths in 2020 worldwide (2). Cancer statistics of China (2015) have reported that HCC is the second and fifth most common tumor-related death in male and female populations, respectively (3). Many factors, such as chronic hepatitis B (and) or C virus infection, aflatoxin B1 contamination, obesity, redundant alcohol assumption, and non-alcoholic fatty liver diseases, have been identified as risky elements for HCC (4, 5). More recently, advances in the treatment of it have been implemented in the clinical employment, including surgery, chemotherapy, radiotherapy, etc. Nevertheless, the present mortality rate of it is still disappointing, with a median survival of roughly 12 months (6–9). Besides, even with technical progression in diagnosis practice in the past decades, the morbidity of it is rising (10). Given the current undesirable situation, further understanding of hepatic tumorigenesis and progression is of importance.

N6-methyladinosine (m6a) modification refers to an insertion of a methyl substituent into the N6 loci of adenosine and is the most prevalent mRNA regulation in eukaryotes (11). M6a modification has been documented to regulate post-transcriptional RNA, affecting RNA stability (12), splicing, and translation (13). In mammalians, three types of proteins have been classified as: eraser, with demethylation ability like FTO and ALKBH5 (14, 15); reader, with recognizing and binding to transcripts like YTHDF1/2/3 and YTHDC1/2 (16, 17); and writer, functioning as methyltransferases like METTL3/14 and WTAP (18), which could reversely regulate m6a modification. All of the 23 m6a-related mRNAs are as following: methyltransferase-like 3 (METTL3), METTL14, WT-associated protein (WTAP), RBM15/RBM15B, KIAA1429, and ZC3H13, FTO and ALKBH5, YTH domain-containing protein 1–2 (YTHDC1/2), YTH domain-containing family member 1–3 (YTHDF1/2/3), and insulin-like growth factor-2 mRNA-binding protein (IGF2BP) family IGF2BP1/2/3 (19). M6a modification has been reported to be involved in many pathological and physiological processes, including cancers (20). For instance, Wei Chong and Aline Kowalski-Chauvel have demonstrated that m6a modification participates in colon cancer and glioma (21, 22). Therefore, our study aims to determine m6a-related lncRNA as a potential prognostic biomarker for HCC and provide a novel vision of possible mechanisms in HCC.

## Material and Methods

### Data Source and lncRNA Identification

Both expression data (lncRNA, mRNA) and clinical data of HCC patients were downloaded from The Cancer Genome Atlas (TCGA, https://portal.gdc.cancer.gov/) website. We integrated the downloaded gene expression profile data with perl script and converted Ensembl ID to gene ID to obtain expression matrix and clinical file. The expression level of m6a gene was extracted from the transcriptome matrix, and 23 m6a-related lncRNAs were found by co-expression analysis using the limma package. According to the expression level of m6a-related lncRNA in the sample, the network relationship file and the node attribute file, the igraph package was used to visualize the co-expression network. Then, the expression data of m6a-related lncRNA and the survival data extracted from the clinical data were combined, and the survival package was used for univariate Cox analysis. The obtained prognosis-related lncRNAs were intersected with differentially expressed lncRNAs between tumor and non-tumor tissues to obtain prognosis-related differential lncRNAs.

### Clustering, Survival Analysis, CD274 (PD-L1) -Related lncRNA Heatmap of TCGA Data

A total of 370 HCC patients were clustered into different clusters by ConsensusClusterPlus R package and finally determined by consensus cumulative distribution function and delta area. Then, survminer package was used to perform survival analysis on different classification groups according to the classification results and survival data, and the survival curve was obtained. The expression of other clinical parameters and prognosis-related lncRNA were extracted, and the heatmap package was used to draw a heat map of the correlation between classification and clinical. Correlation heatmap between CD274 and prognosis-related lncRNAs was performed after differential analysis of CD274 by limma R package. In addition, heatmap between clinical data, including age, gender, grade, stage, tumor, node, metastasis as well as clustering group, and down-/up-regulation of lncRNAs was constructed as well.

### CIBERSOR and Gene Set Enrichment Analysis (GSEA)

According to the gene expression matrix, the cibersort algorithm was used to analyze the immune cell content file and the difference analysis was performed. The filter condition p value of the immune cell infiltration results was set to 0.05, and the vioplot package was used to draw the difference violin diagram and the ggpubr package to draw the boxplot. Differential expression of immune cells including naïve B cells, B cells memory, T cells CD4 memory activated/resting, dendritic cells activated/resting, eosinophils, macrophages M0-2, mast cells activated/resting, monocytes, neutrophils, NK cells activated/resting, T cells CD4 naïve/CD8/follicular helper/gamma delta/regulatory (Treg) by clustering group. Fraction difference, estimate score, immune score, and stromal score were assessed by clustering group as well. Then, GSEA was conducted by GSEA software to explore possible metabolic pathways these lncRNAs were involved in.

### Least Absolute1 Shrinkage and Selection Operator (LASSO) Regression and Risk Score Model Analysis

LASSO regression was performed by R package glmnet and calculated the minimum criteria. Then, TCGA data were divided into training and testing sets, which were used for survival analysis, survival receiver operator characteristic (ROC) curve drawing, risk score, and risk score related heatmap.

### Forest Plot, lncRNA-Related Heatmap, Differential and Survival Analysis of Risk Score Model

Clinical factors, including age, gender, grade, stage, and risk score in both training and testing sets were computed hazard ratio (HR) of survival by both univariate and multivariate analysis. After that, differential expression and survival analysis by clinical factors were performed using low- and high-risk score group. The model validation of clinical grouping was performed with the survival and survminer packages, and the p-value was used to observe whether the model was suitable for patients with different clinical traits, and the ggpubr package was used to draw the heatmap and boxplot of the clinical traits. Moreover, correlation plots between risk score and immune cell as well as differential expression between low- and high-risk score groups were drawn.

### Prognosis-Related Downstream Target Screening and Enrichment Analysis

Finding the miRNAs regulated by AC012073.1 from the Starbase (https://starbase.sysu.edu.cn/) website, and selected the top five microRNAs according to agoexpnum from large to small. The downstream mRNA was searched according to miRTarBase (https://mirtarbase.cuhk.edu.cn/~miRTarBase/miRTarBase_2022/php/index.php) and TargetScan (http://www.targetscan.org/vert_71/). According to the corresponding regulatory relationship, Cytoscape software was used to draw the lncRNA-miRNA-mRNA interaction map. For the obtained mRNA targets, prognostic correlation analysis was performed: merged the files of survival data and expression data, used the survival R package to perform Cox analysis, set the significance filter condition p value to 0.00001, and obtained the prognostic mRNA and the fluctuation range of HR values. Using the plot function to draw a forest graph, set high-risk mRNAs to be represented in red, and low-risk mRNAs to be represented in green. The perl script was used to extract the expression data files and phenotype data files of the target mRNA from the gene expression matrix, and the GSEA enrichment analysis was performed with the GSEA software. Using the same method, the downstream target prediction analysis of all prognosis-related lncRNAs was performed, and the downstream regulated microRNAs and mRNAs were obtained, and the ceRNA network interaction map was drawn according to the regulatory relationship. According to the interaction network, mRNA targets co-regulated by two or more lncRNAs were selected as potential co-acting targets.

### Specimen Collection

A total of 10 pairs specimen (10 specimen of Barcelona Clinical Liver Cancer (BCLC) C stage as experimental group, 10 specimens of BCLC A stage as control group). All patients signed informed consent and the study was approved by the Ethical Committee of The First Affiliated Hospital of Guangxi Medical University (2021(KY-E-336)). Specimens were collected after surgery with approximately 100 mg and stored into EP pipe with 1 ml pre-cooled RNAstore Reagent (cat. No. DP408-02; TIANGEN BIOTECH). Then, specimens were stored in -80°C refrigerator.

### Cell Culture and RT-PCR Assay

Cell lines of Huh7 and Hep3B were purchased from Procell Company and cultured in the DMEM (Dulbecco’s Modified Eagle Medium; cat no.8121587; Gibco) culture with 10% FBS (fetal bovine serum; cat no.2146373; VivaCell) and 1% penicillin and streptomycin (cat no.P1400; Solarbio). Total RNA of specimen and cell line were extracted by NucleoZOL reagent (cat no.740404.200; Gene Company Limited). Reverse transcription was used (TransScript^®^ Uni All-in-One First-Strand cDNA Synthesis SuperMix) for qPCR (One-Step gDNA Removal) (cat no.AU341-02; Transgen). Roche LightCycler 480 machine was employed to perform RT-PCR. FastStart Universal SYBR Green Master (Rox) (cat no.04913914001; Roche) was used for RT-PCR. GAPDH was used as inner control and the relative expressions of lncRNA LOC105377626 were calculated by 2^-△△CT^ method. Primers of GAPDH and LOC105377626 were as follows: (GAPDH) Forward: GCACCGTCAAGGCTG AGAAC, Reverse: TGGTGAAGACGCCAGTGGA, (LOC105377626) Forward: TCTGTGCTGGACTCCTGCTACTC, Reverse: TGCTTCGGATAGAGGTGCTGAGG. The reaction conditions are set as follows: Hold stage: 50°C for 2 min, 95°C for 2 min. Amplification reaction stage: 95°C for 15 s, 60°C for 1 min, a total of 40 cycles. Melt stage: 95°C for 15 s, 60°C for 1 min, 95°C for 30 s, 60°C for 15 s. The melting curve and amplification curve of each gene in the whole reaction process are automatically generated by the instrument.

### RNA Cytoplasmic and Nuclear Separation, Cell Transfection Assays

A total of 5 x 10^6^ cells were collected and total RNA was performed according to the manufacturer’s instructions (cat no.01030165; Invitrogen, Ambion^®^ PARIS™). Reverse transcription and RT-PCR assays were performed according to the above procedures. Then, LOC105377626 expressions in both cytoplasmic and nuclear were calculated separately to distinguish cellular location of LOC105377626. Cells were seeded into 6-plate wells and then transfected with antisense oligonucleotide (100 nM, Ribobio Company) and Lipo6000 reagent (cat no.C0526; Beyotime Biotechnology) in 500 ul DEME volume for 24h. After 48h, cells were digested for further experiments.

### Colony Formation, CCK-8, and EdU Assays

Transfected cells (2000 cell/pore) were seeded into 6-plate wells for colony formation assay. Twenty days later, cells were fixed by 4% paraformaldehyde (cat no. P1110; Solarbio) for 15 min and dyed by 0.1% crystal (cat no.G1064; Solarbio) violet for 10 min. Then, colonies were counted and compared between groups. Transfected cells (1000 cell/pore) were seeded into 96-plate wells for CCK-8 assay. Cells were cultured with a mixture of 10ul of CCK8 reagent (cat no. MA0218; Meilunbio^®^) and 100ul of serum-free DMEM medium for 1h and measured OD values of 450 nm at 0h, 24h, 48h, 72h, 96h, and 120h. Then, proliferation curves were drawn and differences were compared between groups. Transfected cells (3 x 10^4^ cell/pore) were seeded into 96-plate plates for EdU assay (Cell-LightTM EdU Apollo567 *In Vitro* Kit; cat no.C10310-1; Ribobio Company). Procedures were conducted according to the manufacturer’s instructions.

### Transwell and Scratch Assays

In migration assay, transfected cells (1x10^5^cell) were seeded into upper room of Transwell plate (8μm pore size, Corning Costar, USA). Lower room was added 700 μl DMEM with 20% FBS and Transwell plate was cultured for 24h in the incubator at 37°C. Transwell plate was fixed by 4% paraformaldehyde and dyed with 0.1% crystal violet. Then, cells were counted in the microscope and compared between groups. In invasion assay, Transwell plates were added 60 μl Matrigel (cat no.354248; Corning Costar, USA) after the Matrigel were diluted 10 times with DMEM. Then, the Transwell plate was placed into the incubator at 37°C for 1h. The rest of the procedure was conducted the same as in the migration assay. Transfected cells (3 x 10^5^ cell/pore) were seeded into 6-plate wells for 24h. Then, scratch of each pore with “+” was conducted and cells were cultured in DMEM with 1% FBS. After 48h, cells were pictured by the microscope and distances and migration distance were measured for comparison.

### Statistical Analysis

A P ≤ 0.05 was identified as a significant statistical difference in comparing groups. Non-paired T test or Mann Whitney Test was used to compare the differences between two groups. Differential expression, survival analysis, and interaction network were performed by R (version 4.1.1, https://www.r-project.org/) platform. For Kaplan-Meier curves, p-values and hazard ratio (HR) with 95% confidence interval (CI) were generated by log-rank tests and univariate Cox proportional hazards regression. Cox analysis was used to analyze survival-related risk factors. If the HR value was greater than 1, the factor was a risk factor; if HR<1, the factor was a protective factor; if HR=1, the factor had nothing to do with patient prognosis. The confidence interval of the Cox analysis was set as 95%. Unpaired T test was used to compare the results between groups in the *in vitro* cell experiment; Mann Whitney Test was used to compare the differences in lncRNA expression in the tissues of patients with stage A and stage C.

## Results

### Flow Diagram and lncRNA Identification

**Figure 1:**
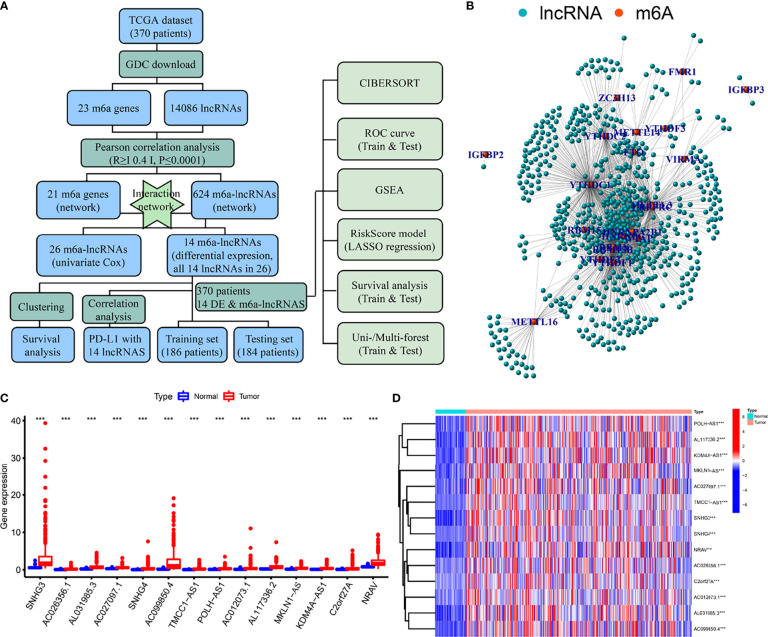
Flow diagram, interaction network, and differential analysis. **(A)** Flow diagram of the present study, **(B)** Interaction network of m6a-related mRNA and lncRNAs, **(C, D)** Box plots and heatmap of differential lncRNAs. *** means P<0.001.

A flow diagram was drawn to exhibit the analyzing process of the present study (**Figure 1A**). Then, interaction network between m6a modification-related mRNAs and lncRNAs was established in **Figure 1B**. Among 23 m6a modification-related mRNAs, 21 of them were enriched in the network, such as *IGFBP2*/*3*, *YTHDC1*/*2*, *METTL3*/*14*/*16*, and *YTHDF1*/*2*/*3*. After that, univariate survival analysis found 26 prognosis-related lncRNAs, with all of them indicating higher risk of survival (all *P*<0.001, HR>1, **Table 1**), including AL158166.1, SNHG3, LINC01224, AL442125.2, *DUXAP8*, *NRAV*, etc. In addition, differential expression identified 14 lncRNAs, such as *SNHG3*, *AC026256.1*, *AL031985.3*, *SNHG4*, *NRAV*, etc., all high in tumor tissues and heatmap visualized their expressions (**Figures 1C, D**). Interestingly, 14 differential lncRNAs were consistently shown in 26 prognosis-related lncRNAs.

**Table 1:** Identification of 26 prognosis-related lncRNAs by univariate analysis.

LncRNA	P value	HR (95% CI)
*AL158166.1*	<0.001	1.876 (1.395−2.523)
*SNHG3*	<0.001	1.069 (1.039−1.099)
*LINC01224*	<0.001	1.869 (1.411−2.475)
*AL442125.2*	<0.001	3.665 (1.943−6.913)
*AC145207.5*	<0.001	1.771 (1.368−2.292)
*AC026356.1*	<0.001	4.256 (2.297−7.889)
*THUMPD3−AS1*	<0.001	1.381 (1.182−1.615)
*AC034229.4*	<0.001	1.987 (1.415−2.791)
*AL031985.3*	<0.001	1.879 (1.547−2.283)
*WAC−AS1*	<0.001	1.095 (1.050−1.142)
*LINC01138*	<0.001	1.400 (1.203−1.628)
*AC010789.1*	<0.001	1.863 (1.362−2.548)
*AC027097.1*	<0.001	2.118 (1.519−2.955)
*SNHG4*	<0.001	1.482 (1.284−1.711)
*AC099850.4*	<0.001	1.145 (1.093−1.199)
*AC026412.3*	<0.001	14.008 (4.327−45.347)
*TMCC1−AS1*	<0.001	2.641 (1.846−3.777)
*DUXAP8*	<0.001	4.712 (2.247−9.884)
*DDX11−AS1*	<0.001	4.586 (2.226−9.450)
*POLH−AS1*	<0.001	2.534 (1.687−3.806)
*AC012073.1*	<0.001	1.412 (1.232−1.619)
*AL117336.2*	<0.001	1.605 (1.347−1.913)
*MKLN1−AS*	<0.001	3.378 (2.285−4.992)
*KDM4A−AS1*	<0.001	2.852 (1.898−4.286)
*C2orf27A*	<0.001	1.623 (1.313−2.006)
*NRAV*	<0.001	1.235 (1.130−1.350)

HR, hazard ratio; 95% CI, 95% confidence interval.

### Clustering, Survival Analysis, and Establishment of CD274 (PD-L1)-Related lncRNA Heatmap

**Figure 2:**
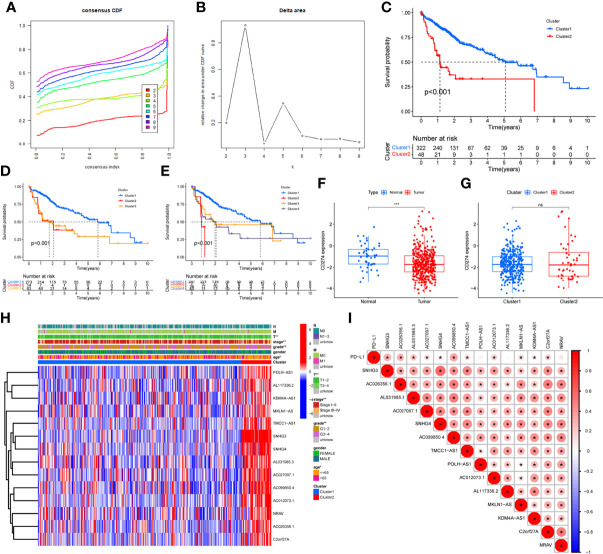
Differential expression, correlation analysis, and survival analysis of lncRNAs. **(A, B)** Consensus cumulative distribution function and delta area plots, **(C–E)** Survival analysis by cluster 2 **(C)**, cluster 3 **(D)**, cluster 4 **(E)**, **(F, G)** Differential analysis of CD274 by tissue type **(F)** and cluster **(G)**, **(H)** LncRNAs and clinical factors-related heatmap, **(I)** Correlation analysis between lncRNAs and PD-L1. The symbols of “ns” means P>0.05. * means P<0.05. ** means P <0.01. *** means P<0.001.

Clustering was divided into eight groups labeled2 through 9. According to consensus cumulative distribution function and delta area (**Figures 2A, B**), Cluster 2, Cluster 3, and Cluster 4 drew the survival plots (**Figures 2C–E**, all *P*<0.001) and only two of the groups were picked for further analysis concerning patient numbers in each group. Then, differential expression of CD274 was analyzed and found only in tissue type but not cluster (**Figures 2F, G**, *P<0.001, P>0.05*). Then, expression and correlation heatmaps were established to indicate correlations between clinical factors and PD-L1 with these survival-correlated lncRNAs (**Figures 2H, I**). PD-L1 was correlated with 11 of these lncRNAs (**Figure 2I**, all *P* ≤ 0.05).

### CIBERSORT and GSEA

**Figure 3:**
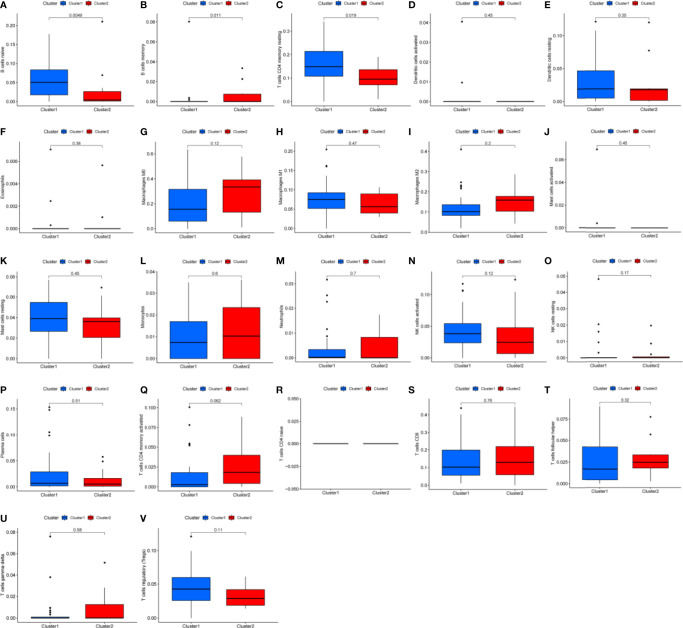
Box plots of differential analysis by immune cells. **(A–V)** Box plots of differential analysis of B cell, T cell, dendritic cell, eosinophils, macrophages, mast cells, monocytes, neutrophils, natural killer cell, and plasma cells, respectively.

**Figure 4:**
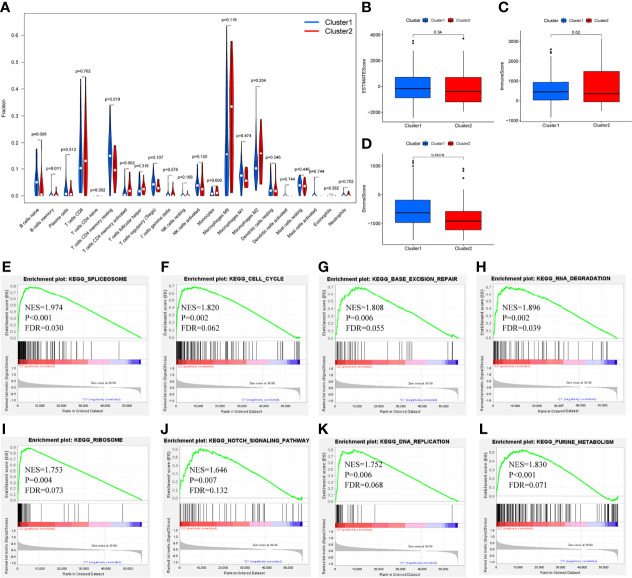
Differential fraction, box plots and enrichment analysis. **(A)** Differential fraction comparison of immune cells, **(B–D)** Box plots of estimatescore, immunescore, and stromalscore by clusters, **(E–L)** Enrichment analysis of KEGG pathways of lncRNAs-related.

Differential analysis was conducted in immune cells by cluster group. Among them, three types of immune cells demonstrated differential expression, with a higher level in naïve B cells and T cells CD4 memory resting and lower level in B cells memory (**Figures 3A–C**, **4A**, all *P*<0.05). Differential expression did not show in other types of immune cell (**Figures 3D–V**, all *P*>0.05). Differential score was assessed and only showed a difference in stromal score but did not ESTIMATE score and immune score (**Figures 4B–D**, *P*=0.34, 0.62, 0.018). On the other hand, exploration of possible mechanisms by GSEA showed that these lncRNAs participated in spliceosome, cell cycle, base excision repair, RNA degradation, ribosome, Notch signaling pathway, DNA replication, purine metabolism, etc. (**Figures 4E–L**, all *P*<0.05, false discovery rate<0.25).

### LASSO Regression and Risk Score Model Analysis

**Figure 5:**
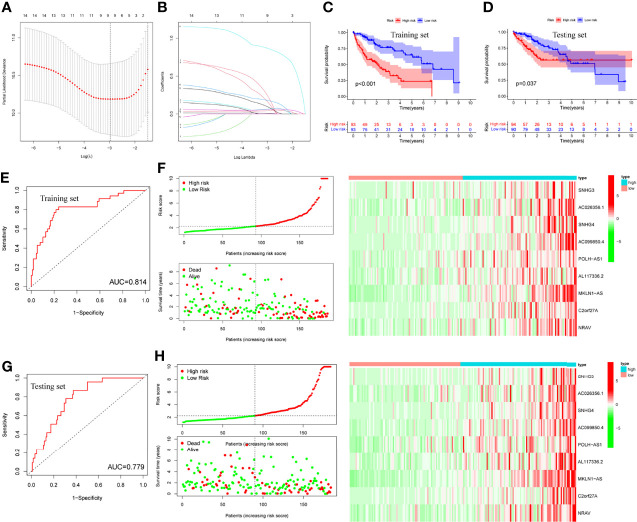
LASSO regression, survival plots and risk score models. **(A, B)** LASSO regression plots, **(C, D)** Survival analysis of training and testing sets by risk groups, respectively, **(E, F)** Risk score model, ROC curve and heatmap of training set, **(G, H)** Risk score model, ROC curve and heatmap of testing set.

LASSO regression was performed and identified the minimum criteria (**Figures 5A, B**). It has been applied for the optimal choice to avoid overfitting as well as to discern the most meaningful model for predicting survival outcome. Then, 370 HCC patients were divided into training set (186 patients) and testing set (184 patients). Survival analysis of two sets was conducted and both indicated significant differences (**Figures 5C, D**, *P*<0.001, *P*=0.037). Then, survival ROC curves and risk score models, including risk score, survival status, and heatmap, were calculated and visualized, with both sets showing high AUC (training: 0.814, testing: 0.779, **Figures 5E–H**).

### LncRNA-Related Heatmap, Differential, and Survival Analysis of Risk Score Model

**Figure 6:**
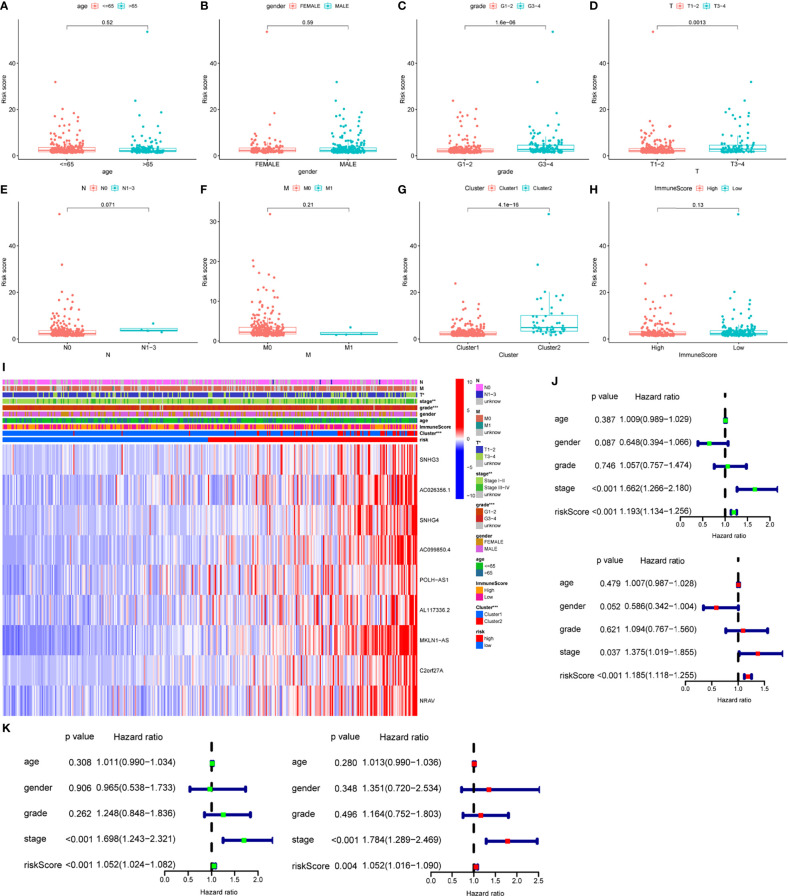
Box plots, heatmap and forest plots of lncRNAs and clinical factors-related. **(A–H)** Box plots of risk score by age, gender, grade, tumor, node, metastasis, cluster, immunescore, respectively, **(I)** Heatmap of lncRNAs, clinical factors, immunescore and cluster-related, **(J, K)** Forest plots of univariate and multivariate analysis in training and testing sets, respectively. ** means P<0.01. *** means P<0.001.

**Figure 7:**
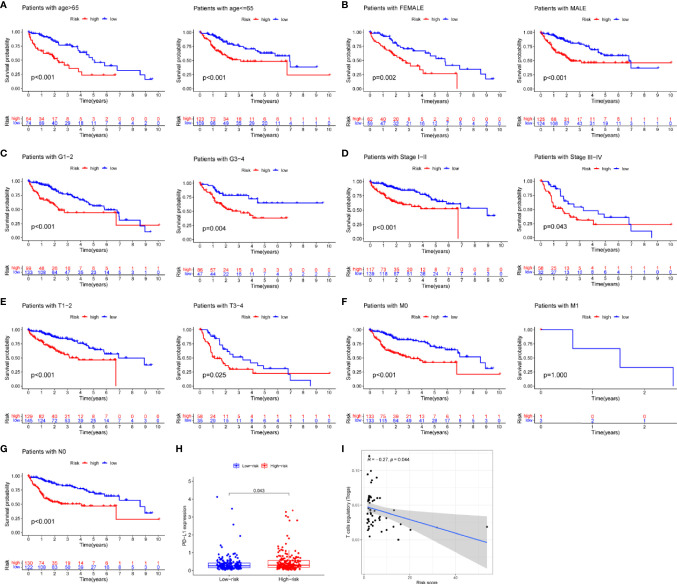
Survival analysis, box plots and correlation plots of clinical factors. **(A–G)** Survival analysis by age, gender, grade, stage, tumor and metastasis and node, respectively, **(H)** Box plot of PD-L1 expression by risk groups, **(I)** Correlation plot between regulatory T cells and risk scores.

Risk score was assessed by clinical factors and showed a different risk score in grade, T, and cluster(**Figures 6C, D, G**, all *P*<0.05), but did not in age, gender, N, M, and immunesocre **(** **Figures 6A, B, E, F, H**, all *P*>0.05). Then, heatmap was established to indicate lncRNA expression, clinical factors, risk score, immune score, and cluster (**Figure 6I**). Moreover, both univariate and multivariate analysis were performed and found stage and risk scores steadily influenced clinical outcomes (all *P*<0.05, HR>1) but not for age, gender, and grade in training and testing sets (**Figures 6J, K**). After that, survival analysis by differential clinical factors was performed and demonstrated a significant difference in age (<65, >65 years old), gender (male, female), G (G1-2, G3-4), T (T1-2, 3-4), stage (I-II, III-IV), M0, and N0 (**Figures 7A–G**) but did not in M1 group. PD-L1 differential expression analysis found that the high-risk group showed higher PD-L1 expression (**Figure 7H**,*P*=0.043). Correlation analysis demonstrated that T cells regulatory was positively correlated to risk score (**Figure 7I**, r=-0.27, *P*=0.044).

### Prognosis-Related Downstream Target Gene Screening and Enrichment Analysis

**Figure 8:**
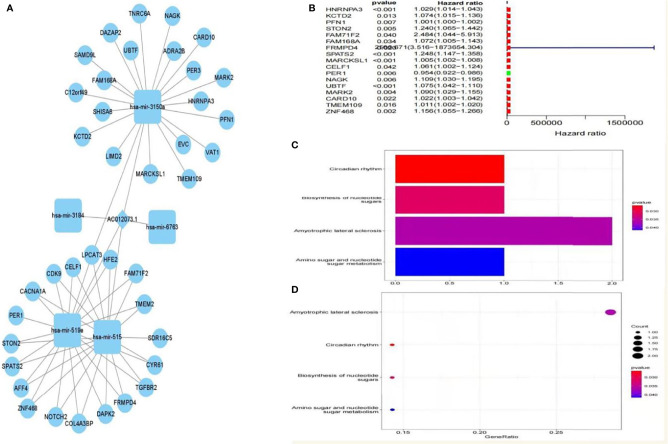
Prognosis-related downstream target gene screening and enrichment analysis. **(A)** ceRNA interaction regulatory network based on AC012073.1, **(B)** Forest plot of prognosis-related downstream genes, **(C)** Bar graph of the results of KEGG enrichment analysis of prognosis-related downstream genes, **(D)** Bubble plot of KEGG enrichment analysis results of prognosis-related downstream genes.

A total of five miRNAs regulated by lncRNA AC012073.1 were obtained by prediction, of which three miRNAs regulated 39 mRNAs, and an interaction network diagram was drawn according to their regulatory relationships (**Figure 8A**). Cox regression was used for prognostic analysis, and a total of 17 prognostic-related mRNAs were screened out, all of which had p-values less than 0.01 and were all high-risk genes (**Figure 8B**). KEGG enrichment analysis showed that these genes were mainly involved in biological activities such as SPLICEOSOME, PYRIMIDINE_METABOLISM, RNA_DEGRADATION, PURINE_METABOLISM and CELL_CYCLE (**Figures 8C, D**). The downstream targets of prognosis-related lncRNAs were predicted, gene targets that were jointly regulated by more than two lncRNAs were screened, and two target genes, MYLIP and HDGF, were obtained **(** **Supplemental Figure S1** **)**.

### Validation of Differential Expression and *In Vitro* Experiments

**Figure 9:**
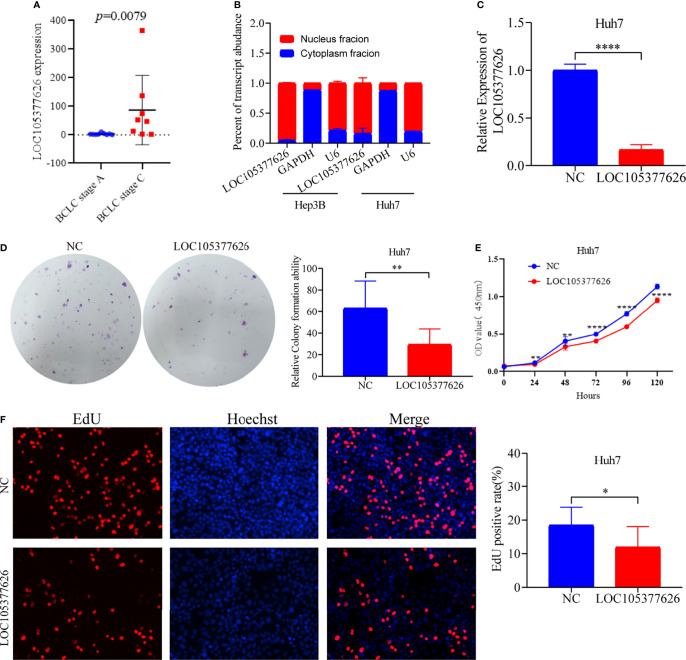
Differential expression, cellular location, and *in vitro* experiments of LOC105377626. **(A)** Differential expression of LOC105377626 between TNM stage A and stage C, **(B)** Cellular location results of LOC105377626 in Hep3B and Huh7 cell lines, **(C)** Relative expression of LOC105377626 in control and knockdown groups, **(D–F)** Results of colony formation, CCK-8 and EdU assays between LOC105377626 control and knockdown groups. * means P<0.05. ** means P<0.01.**** means P<0.0001.

**Figure 10:**
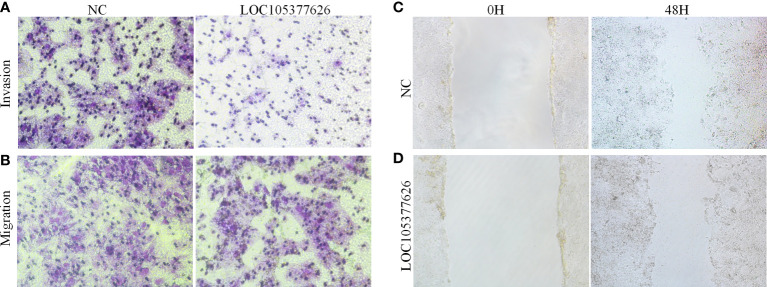
Transwell and scratch assays results of LOC105377626 knockdown. **(A, B)** Results of Transwell (with and without Matrigel) assays between LOC105377626 control and knockdown groups, respectively, **(C, D)** Results of scratch assay between LOC105377626 control and knockdown groups.

Then, sequence analysis found that AC012073.1 includes an uncharacterized sequence, namely LOC105377626. After removing values that deviate too much, differential expression found that BCLC stage C had higher levels than BCLC stage A (**Figure 9A**, *P*=0.0079). Assay of RNA cytoplasmic and nuclear separation suggested that LOC105377626 was mainly congregated in nucleus compared to cytoplasm (Hep3B: foldchange=19.875, Huh7: foldchange=5.238, **Figure 9B**). After that, LOC105377626 was down-regulated by antisense oligonucleotide (**Figure 9C**, *P ≤* 0.001**)**. We used colony formation, CCK-8, and EdU assays and found that down-regulated LOC105377626 demonstrated reduced colony formation ability (**Figure 9D**, foldchange=2.148, *P ≤* 0.01) and growth ability (**Figures 9E, F**, foldchage=1.545, all *P ≤* 0.05). Moreover, Transwell (with and without Matrigel) and scratch assays also consistently suggested that down-regulated LOC105377626 demonstrated reduced invasion (**Figure 10A**, *P ≤* 0.05) and migration abilities (**Figures 10B, C**, *P ≤* 0.05).

## Discussion

The m6a modification is one of the most common epigenetic alterations of mRNAs and lncRNAs. It has also been implicated in the regulation of tumor RNAs (23, 24). Furthermore, m6a regulators, including erasers, readers, and writers, have been reported to function in m6a deposition on mammalian nuclear RNAs (18). Of these, METTL3 functions as a catalytic substrate, whereas METTL14 functions in RNA-binding (25). By contrast, FTO and ALKBH5 are RNA demethylases that can remove the m6a modification (14, 26). Several other m6a readers have also been reported to be involved in RNA stability, splicing, translocation, translation, circRNA ring formation, and RNA–protein interactions (25). A previous study has reported that the aberrant expression of m6a modification proteins can facilitate tumor development and progression (27). For instance, Guo et al. and Tian et al. reported that the m6a modification can promote the progression of pancreatic ductal adenocarcinoma, which involves the expression of m6a-related mRNAs such as *METTL14*, *IGFBP2*, and *ALKBH5* (28–30). Li et al. demonstrated that *FTO*, an m6a RNA demethylase, has an oncogenic role; it can promote leukemogenesis and inhibit leukemia cell differentiation in acute myeloid leukemia (31). In another study, Sun et al. demonstrated that the aberrant expression of m6a RNA modulators was significantly associated with tumor progression and survival of patients with kidney renal papillary cell carcinoma (32). We found that these mRNAs (*IGFBP2*/*3*, *YTHDC1*/*2*, *METTL3*/*14*/*16*, *YTHDF1*/*2*/*3*, *RBM15*) were involved in the m6a-related lncRNA network, and of these, 14 lncRNAs were differentially expressed and associated with the clinical outcome of HCC. Therefore, we conclude that m6a-related mRNAs, especially *METTL14*, *IGFBP2*, and *FTO*, can influence the clinical outcome of HCC by altering tumor cell proliferation and survival. Further *in vitro* and *in vivo* experiments are needed to confirm these findings.

LncRNAs, non-coding RNAs with lengths of > 200 nucleotides, regulate a variety of processes, including tumor cell proliferation and metastasis (33, 34). In addition, they modulate gene expression by regulating epigenetic, transcriptional, and posttranscriptional events, which control cell proliferation, differentiation, migration, and survival (35–37). The aberrant expression of lncRNAs has been demonstrated in a variety of tumors (38). For instance, Ma et al. reported that LBX2-AS1, an m6a-related lncRNA, was highly expressed in colorectal cancer, where it was an indicator of poor prognosis (39). Qiu et al. identified 27 differentially expressed (24 up-regulated, 3 down-regulated) lncRNAs in clear cell renal cell carcinoma (40). We identified 26 differentially expressed mRNAs in HCC, although no intersections were identified among them. In enrichment analysis, the Notch signaling pathway was the predicted pathway. Moreover, naïve B cell infiltration was observed. Consistent with the results of Qiu et al., who constructed a risk score model for the survival prediction of renal cell carcinoma using m6a-related lncRNAs, we identified stage and the risk score as the influencing factors of the prognosis of HCC.

In osteosarcoma, ALKBH5-mediated m6a demethylation of lncRNA PVT1, an oncogene, was up-regulated, where it correlated with clinical stage, tumor size, and poor prognosis (41). In colon cancer, Tan et al. identified 10 m6a-related mRNAs and 4060 differentially expressed lncRNAs in TCGA database and used three subgroups for consensus clustering (42). These authors further conducted GSEA and identified pathways, such as oxidative phosphorylation, respiratory electron transport, the citric acid cycle, and respiratory election transport, but none of these pathways were identified in this study. Furthermore, there were no intersections of prognosis-related lncRNAs between the two studies. Li et al. reported mRNA signatures of lung adenocarcinoma using TCGA database and identified 19 m6a-related RNAs, including *YTHDF2*, *ZC3H13*, *YTHDF1*, *FTO*, *WTAP*, *METTL14*, *ALKBH3*, and *ALKBH5* (43). Most of these RNAs were also identified in the interaction network in our study, and their GSEA results were consistent with our GSEA results. These findings indicate that prognosis-associated lncRNAs interact with mRNAs in HCC. However, further studies are needed to confirm these findings.

Risk score models of several prognostic signatures have been widely applied in the survival prediction of glioma (44), colon cancer (21), pancreatic ductal adenocarcinoma (45), kidney papillary cell carcinoma (32), and lung adenocarcinoma (43). This study constructed risk score models to predict the clinical outcome of HCC patients using prognosis-related lncRNAs, similar to the risk score models of other types of cancer. Our results indicated that the risk score model is a useful tool for predicting the survival outcome of patients.

Among the prognosis-related lncRNAs in HCC, SNHG3, SNHG4, and NRAV have been reported to function in bladder cancer (46), non-small cell lung cancer (47), osteosarcoma (48), colorectal cancer (49), breast cancer (50), gastric cancer (51), and glioma (52) through interactive molecular pathway studies. However, other lncRNAs, such as AC099850.4 (53), MKLN1-AS (54, 55), and C2orf27A (56) have been reported in HCC. POLH-AS1 POLH-AS1 have not been reported in HCC and we detected its expression of HCC tissues in BCLC stage A and BCLC stage C by RT-PCR. There was no significant difference in its expression between the two different stages. This study focused on LOC105377626 and revealed its functions in HCC. *In vitro* experiments indicated that LOC105377626 functions as an oncogene in HCC cell lines, where it localized to the nucleus. The role of AC012073.1 in esophageal squamous cell carcinoma has been reported in only one risk score model study (57), where AC012037.1 was found to bind to miR-93. It also has prognostic value and participates in the competitive endogenous RNA network in cancer. Therefore, more studies are needed to explore the role of AC012037.1 (LOC105377626) in cancer.

There were some limitations in this study. Firstly, *in vitro* and *in vivo* experiments that focus on the metabolic pathways should be performed. Secondly, a larger cohort is needed to confirm our findings. Thirdly, additional clinical factors should be identified and more follow-up data should be analyzed.

## Conclusion

Through the TCGA database, we identified 14 differentially expressed and 26 prognosis-related m6a-associated mRNAs in HCC. Correlation analysis was used to construct the mRNA–lncRNA network, and consensus clustering revealed two subgroups for further analysis. LASSO regression determined the risk score model, which was patient survival by prognosis-related lncRNAs. The immune infiltration of naïve B cells, CD4 memory resting T cells, and memory B cells was variable in the different cluster groups. GSEA indicated that lncRNAs have roles in spliceosome dynamics, the cell cycle, base excision repair, RNA degradation, ribosome dynamics, the Notch signaling pathway, DNA replication, and purine metabolism. Moreover, *in vitro* experiments of the uncharacterized sequence LOC105377626, a member of AC012073.1, indicated that it functions as an oncogene in HCC. Further mechanistic studies are needed on AC012073.1 (LOC105377626).

## Data Availability Statement

The original contributions presented in the study are included in the article/**Supplementary Material**. Further inquiries can be directed to the corresponding authors.

## Ethics Statement

The studies involving human participants were reviewed and approved by Ethical Review Committee of First Affiliated Hospital of Guangxi Medical University. The patients/participants provided their written informed consent to participate in this study.

## Author Contributions

Conceptualization, XW, ZD, and HJ; Methodology, XW, ZD, and HJ; Validation, XW; Formal Analysis, XW; Investigation, XW, ZD, XL, and XR; Resources, NQ, XS, and LP; Writing – Original Draft, XW, ZD, and NQ; Writing Review & Editing XW, SQ, and HJ; Funding Acquisition SQ and,HJ; Supervision SQ and HJ. All authors contributed to the article and approved the submitted version.

## Funding

This work was supported in part by the National Natural Science Foundation of China (81960119) and the Scientific Research Project of Guangxi Health Commission (No. z2015196).

## Conflict of Interest

The authors declare that the research was conducted in the absence of any commercial or financial relationships that could be construed as a potential conflict of interest.

## Publisher’s Note

All claims expressed in this article are solely those of the authors and do not necessarily represent those of their affiliated organizations, or those of the publisher, the editors and the reviewers. Any product that may be evaluated in this article, or claim that may be made by its manufacturer, is not guaranteed or endorsed by the publisher.

## Supplementary Material

Supplementary Figure 1 Network interaction map of 14 lncRNAs acting on common mRNA targets.
