# Supplementary figures and images for: Establishment of Prognostic Signatures of N6-Methyladenosine-Related lncRNAs and Their Potential Functions in Hepatocellular Carcinoma Patients
Source: Front Oncol. 2022 Jun 6;12:865917. doi: 10.3389/fonc.2022.865917 (PMC9207396; doi:10.3389/fonc.2022.865917)

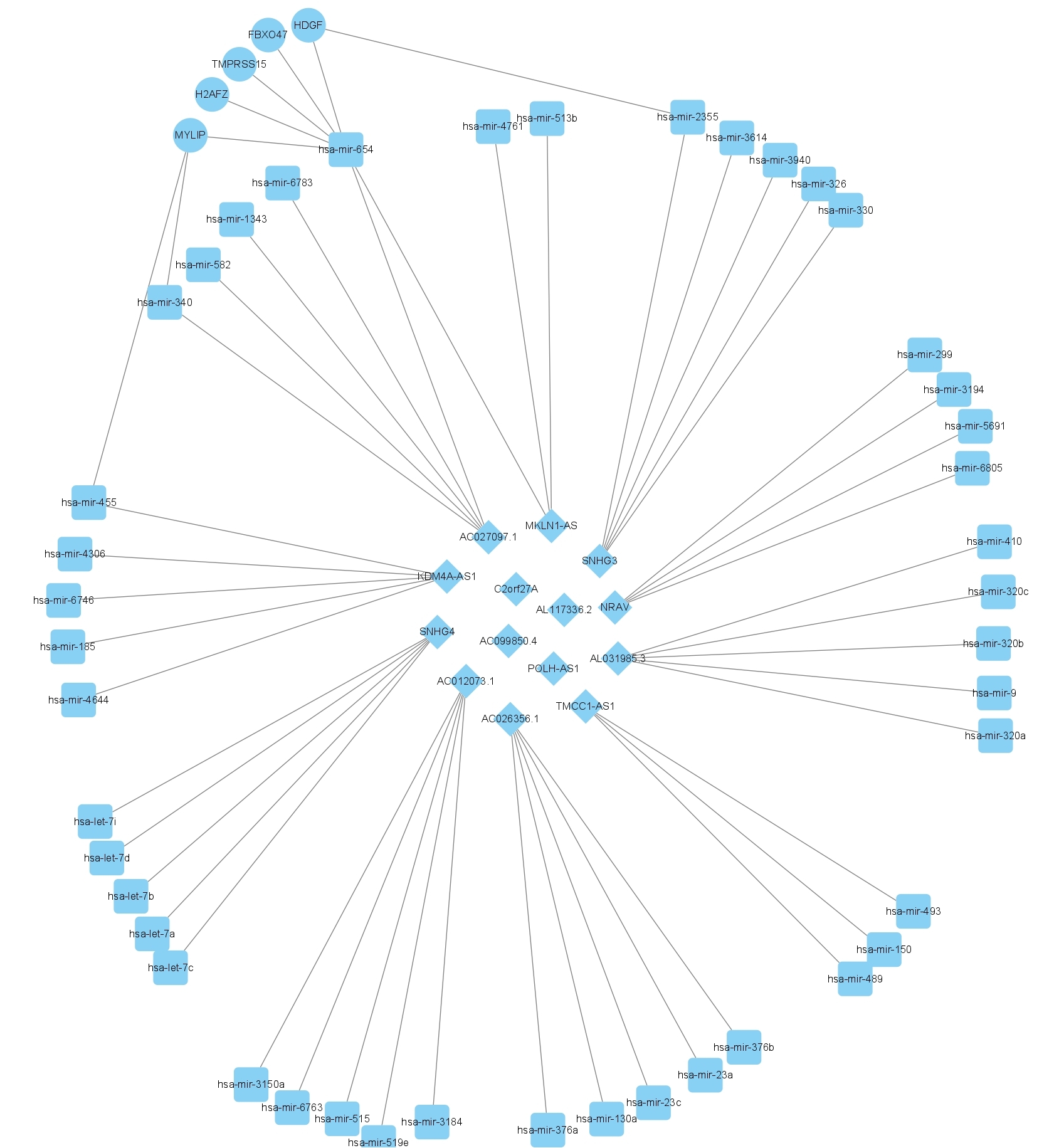

Supplement: Supplementary Figure 1 — Network interaction map of 14 lncRNAs acting on common mRNA targets. [file Image_1.tif]

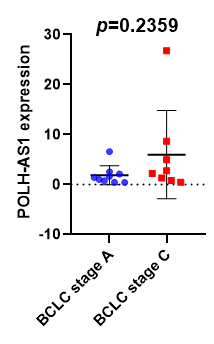

Supplement: Supplementary file 2 [file Image_2.tif]
